# Detection of extended spectrum β-lactamase in *Pseudomonas* spp. isolated from two tertiary care hospitals in Bangladesh

**DOI:** 10.1186/1756-0500-6-7

**Published:** 2013-01-05

**Authors:** Shahanara Begum, Md Abdus Salam, Kh Faisal Alam, Nurjahan Begum, Pervez Hassan, Jalaluddin Ashraful Haq

**Affiliations:** 1 Department of Microbiology, Green Life Medical College, Dhaka, Bangladesh; 2 Department of Microbiology, Rajshahi Medical College, Rajshahi, 6000, Bangladesh; 3 Department of Microbiology, Pabna Medical College, Pabna, Bangladesh; 4 Institute of Biological Sciences, University of Rajshahi, Rajshahi, 6205, Bangladesh; 5 Department of Microbiology, Ibrahim Medical College, Dhaka, 1000, Bangladesh

**Keywords:** *Pseudomonas* spp, Antimicrobial susceptibility, ESBL

## Abstract

**Background:**

Extended spectrum ß-lactamases (ESBLs) represent a major group of lactamases responsible for resistance, mostly produced by gram-negative bacteria, to newer generations of ß-lactam drugs currently being identified in large numbers worldwide. The present study was undertaken to see the frequency of ESBL producing *Pseudomonas* spp. isolated from six hundred clinical specimens (wound, pus, aural, urine, sputum, throat and other swabs) collected over a period of three years from two tertiary care hospitals in Bangladesh.

**Findings:**

Aerobic bacterial culture was performed on aseptically collected swabs and only growth of *Pseudomonas* was considered for further species identification and ESBL production along with serotyping of *Pseudomonas aeruginosa*. Antimicrobial susceptibility testing was carried out using the Kirby-Bauer agar diffusion method and ESBL production was detected on Mueller Hinton agar by double-disk synergy technique using Amoxicillin-Clavulanic acid with Ceftazidime, Cefotaxime, Ceftriaxone and Aztreonam. Culture yielded 120 *Pseudomonas* spp*.* and 82 of them were biochemically characterized for species. *Pseudomonas aeruginosa* was found to be the predominant (90.2%) species. Of 82 isolates tested for ESBL, 31 (37.8%) were ESBL positive with 29 (93.5%) as *Pseudomonas aeruginosa,* the remaining 2 (6.5%) were *Stenotrophomonas maltophilia* and *Ralstonia pickettii.* Antibiogram revealed Imipenem as the most effective drug (93.3%) among all antimicrobials used against *Pseudomonas* spp. followed by Aminoglycosides (63.7%).

**Conclusion:**

ESBL producing *Pseudomonas* spp. was found to be a frequent isolate from two tertiary care hospitals in Bangladesh, showing limited susceptibility to antimicrobials and decreased susceptibility to Imipenem in particular, which is a matter of great concern.

## Background

The worldwide emergence of multi-drug resistant bacterial strains is a growing concern, especially infections caused by *Pseudomonas* spp. and *P. aeruginosa* in particular. *P. aeruginosa* is an opportunistic pathogen with innate resistance to many antibiotics and disinfectants including anti-pseudomonal Penicillins, Ceftazidime, Carbapenems, Aminoglycosides and Ciprofloxacin [1]. Infections due to *P. aeruginosa* are seldom encountered in healthy adults; but in the last two decades, the organism has become increasingly recognized as the etiological agent in patients with impaired immune defenses [2]. Pseudomonads are more versatile than *Enterobacteriaceae* in acquiring drug resistance by various mechanisms. The production of extended-spectrum beta-lactamases (ESBLs) confers resistance at various levels to expanded spectrum Cephalosporins [3]. These enzymes are encoded by different genes located on either chromosomes or plasmids [4]. ESBL-producing bacteria may not be detectable by the routine disk diffusion susceptibility test, leading to inappropriate use of antibiotics and treatment failure.

Several different methods have been suggested for the detection of ESBLs in clinical isolates [5], such as disk approximation or double disk synergy, modified double disc test (MDDT), CLSI phenotypic confirmatory method, E-test ESBL strips, three dimensional test, Vitek system, etc. While each of these tests has merit, none are able to detect all of the ESBLs encountered. Disk approximation or double disk synergy is one of the currently available and most widely used techniques for the detection of ESBLs [6].

Although bacterial resistance to the beta-lactam drugs and the mechanisms leading to this resistance have become a primary focus for clinicians and researchers, until recently, only a few studies have been carried out to detect ESBL bacteria in Bangladesh. Further, routine ESBL phenotype screening is not yet practiced in Bangladesh. The present study was conducted with an aim to detect the prevalence of ESBL-producing *Pseudomonas* spp. isolated from clinical samples of two tertiary care hospitals in Bangladesh.

## Findings

### Materials and methods

#### Materials

The study examined 600 swabs including from wounds (n =200), pus (n =110), urine (n =100), aural (n =80), sputum (n =50), throat (n =50), umbilicus and conjunctiva (n =10) taken from patients of different ages and sex attending at Rajshahi Medical College Hospital (RMCH) and BIRDEM Hospital, Dhaka, Bangladesh from July 2000 to September 2003. Laboratory works were performed at the Microbiology laboratory of Rajshahi Medical College, BIRDEM hospital and the Molecular Biology laboratory of the Institute of Biological Sciences (IBSc), Rajshahi University. The study was ethically approved by the Ethical Review Committee of the Institute of Biological Sciences (IBSc), Rajshahi University and written informed consent was obtained from patients or a legal guardian in the case of minors.

#### Culture and identification of species and serotypes

Following aseptic collection, swabs were routinely inoculated onto Blood and MacConkey agar media. The plates were incubated overnight aerobically at 37°C and then checked for bacterial growth. *Pseudomonas* spp. were identified by their colony morphology, staining characters, pigment production, motility and other relevant biochemical tests as per standard methods of identification. Cetrimide agar medium was used as selective media for subculturing *Pseudomonas* spp. Isolates were categorized into different species based on their distinct biochemical and pigment production characteristics [7] and serotyping of *Pseudomonas aeruginosa* was done using commercially available (Denka Seiken Co. Ltd., Japan) polyvalent I, II and III group specific antisera against 14 O antigens.

#### Antimicrobial susceptibility testing

Antimicrobial susceptibility testing of *Pseudomonas* spp. was done by the Kirby-Bauer agar diffusion method using *P. aeruginosa* ATCC 27853 as the control strain. Commercially available (Hi-Media) antimicrobial disks of Piperacillin (PIP 100 μg), Amikacin (AMI 30 μg), Carbinicillin (CARB 100 μg), Ceftazidime (CAZ 30 μg), Ceftriaxone (CRO 30 μg), Cefotaxime (CTX 30 μg), Tetracycline (TET 30 μg), Gentamycin (GEN 10 μg), Ciprofloxacin (CIP 5 μg), Tobramycin (TOB 10 μg), Imipenem (IMP 10 μg) and Netilmycin (NET 30 μg) were used on Mueller Hinton agar (MHA, Hi-Media) to test susceptibility. Zone of inhibition was recorded as *Sensitive* or *Resistant* according to CLSI guidelines [8].

#### Detection of ESBL by double disc diffusion synergy method

ESBL production in *Pseudomonas* spp. was detected by double disk synergy test (DDST) as described by Jarlier [9]. Mueller Hinton agar was inoculated with standardized inoculum (corresponding to 0.5 McFarland tube) using sterile cotton swab. An Augmentin (20 μg Amoxicillin and 10 μg of Clavulanic acid- AMC) disk was placed in the center of the plate and test disks of 3rd generation Cephalosporins (Ceftazidime- CAZ 30 μg, Ceftriaxone-CRO 30 μg, Cefotaxime-CTX 30 μg) and Aztreonam (ATM 30 μg) disks were placed at 20 mm distance (center to center) from the Amoxicillin-Clavulanic acid disk prior to incubation. The plate was incubated overnight at 35°C. Enhancement of the zone of inhibition of any one of the four drug disks toward Amoxicillin-Clavulanic acid suggested the presence of extended-spectrum beta-lactamases.

## Results

Table 1 shows the frequency distribution of *Pseudomonas* spp*.* with the number of ESBL-positive cases. Of 82 strains of *Pseudomonas* spp*.* tested for ESBL, 31 (37.8%) were found as ESBL-positive with the highest frequency (75%) from urine, followed by throat (50%), pus (35%), aural (33.3%) wound (26.3%) and sputum (25%).

**Table 1:** **Rate of detection of *Pseudomonas *spp. and ESBL-positive strains from clinical specimens**

**Specimens (n)**	***Pseudomonas *spp*. *n (%)**	**ESBL-positive n (%)**
Wound swab (200)	19 (9.5)	5 (26.3)
Pus (110)	20 (18.2)	7 (35)
Aural swab (80)	18 (22.5)	6 (33.3)
Urine (100)	12 (12)	9 (75)
Sputum (50)	8 (16)	2 (25)
Throat swab (50)	4 (8)	2 (50)
Others (10)	1 (10)	0 (0)
Total	82	31 (37.8)

Distribution of *Pseudomonas* species and serotypes of *Pseudomonas aeruginosa* with ESBL positive strains are shown in Table 2. Of 120 *Pseudomonas* isolates, 82 were identified at the species level based on their distinct biochemical and pigment production characteristics [7]. *Pseudomonas aeruginosa, Pseudomonas fluorescens, Stenotrophomonas maltophilia, Ralstonia pickettii* and *Pseudomonas alkaligenes* were detected at a frequency of 74 (90.2%), 2 (2.45%), 2 (2.45%), 2 (2.45%) and 2 (2.45%) respectively. Of 74 *Pseudomonas aeruginosa* isolates, 29 (39.2%) were ESBL positive. Serotyping of *Pseudomonas aeruginosa* revealed 25 as A,C,H,I,L (polyvalent group I), 17 as B,J,K,M (polyvalent group II) and 32 as D,E,F,G,N (polyvalent group III).

**Table 2:** ***Pseudomonas *species and serotypes of *Pseudomonas aeruginosa *with ESBL positivity**

**Species**	**Number**	**ESBL-positive n (%)**
*P. aeruginosa*	74	29 (39.2)
Serotypes- A,C,H,I,L −25		
B,J,K,M −17		
D,E,F,G,N-32		
*P. fluorescens*	2	0
*S. multophilia*	2	1 (50)
*R. pickettii*	2	1 (50)
*P. alkaligenes*	2	0
Total	82	31 (37.8)

Antimicrobial susceptibility pattern revealed that Imipenem was the most effective drug against *Pseudomonas* spp. with susceptibility of 93.3%, followed by Tobramycin (66.7%), Amikacin (63.7%) and Gentamycin (60%). Susceptibility of *Pseudomonas* spp. to 3rd generation Cephalosporins ranged from 43.3 to 46.7% and Aminoglycosides had better efficacy than 3rd generation Cephalosporins (Table 3).

**Table 3:** **Antimicrobial susceptibility pattern of *Pseudomonas *spp**

**Antimicrobial drugs**												
Susceptibility%	PIP	CARB	CAZ	CRO	CTX	CIP	TET	AMI	GEN	TOB	NET	IMP
	58	43.3	46.7	46.4	43.3	40	32.5	63.7	60	66.7	53.3	93.3

## Discussion

ESBL-producing organisms pose unique challenges to clinical microbiologists, clinicians, infection control professionals and scientists engaged in finding new antibacterial molecules. ESBL-producing strains are usually found in those hospitals where antibiotic use is frequent and the patients are in critical condition. In the present study, 120 *Pseudomonas* spp*.* were isolated from 600 bacterial cultures with *Pseudomonas aeruginosa* as the most frequent (90.2%) species accounting for 18% of clinical cases. The prevalence of *P. aeruginosa* is consistent with the findings of Wiblin (1997), who documented 16% prevalence for various infections [10].

The rates of ESBL-positive *Pseudomonas* spp*.* (37.8%) and *Pseudomonas aeruginosa* (39.2%) found in our study were in accordance with similar studies conducted in Bangladesh and other South East Asian countries [3,4,11-13], although low detection rates of 3.7% to 7.7% were noted in studies conducted by others [14,15]. Infection patterns, hospital infection control measures and antibiotic policy are all important considerations for variation of detection rates in different hospitals. Of note, we also found two ESBL-negative *P. fluorescens* species, and a recent study has shown that these species produce metallo-beta-lactamase, another very important beta-lactam inhibitor that can act as a reservoir of multidrug resistance element that may be transferred to successful *P. aeruginosa* clones [16].

The distribution of ESBL-positive *Pseudomonas* spp*.* in different samples and its resistance to 3rd generation Cephalosporins is comparable to the findings of Aggarwal et al. [4]. This resistance is due to the hydrolysis of the β-lactam ring by the action of β-lactamase enzymes. Other mechanisms for drug resistance to the β-lactam group of antibiotics are loss of outer membrane protein, production of class C AmpC β-lactamases and altered target sites. ESBL enzymes are inhibited by β-lactamase inhibitors, particularly Clavulanic acid and Sulbactam. Hence the use of β-lactam/β-lactamase inhibitor combination therapy may be an alternative to 3rd generation Cephalosporins, but the effect of this combination varies depending on the subtype of ESBL present [17].

Imipenem was found to be the most efficacious drug against *Pseudomonas* spp. in our study, which is in accordance with findings of Ullah et al. [13]; however, notably in our study, Imipenem underperformed compared to the 100% susceptibility found in ESBL-producing gram-negative isolates, including *P. aeruginosa*, in different studies [13,18]. Decreased susceptibility to Imipenem is a matter of great concern for treating infections caused by *Pseudomonas aeruginosa* and indicates the urgent need for improved infection control strategies.

## Conclusion

ESBL-producing *Pseudomonas* spp., and *P. aeruginosa* in particular, were found to be frequent isolates from two tertiary care hospitals in Bangladesh with limited susceptibility to antimicrobials. Further, decreased susceptibility to Imipenem is a matter of great concern as it is the drug of choice in the treatment of Pseudomonad infection.

## Competing interests

The authors declare that they have no competing interests.

## Authors’ contributions

SB conceived the research idea and performed the laboratory work. KFA and NB helped in sample collection and preparation of the manuscript. JAH and PH helped in designing the study and supervision of the work. MAS contributed intellectual thought, final revision and editing of the manuscript. All authors have read and approved the submitted version of manuscript.
